# The Scenario of Acute Poisoning in Jashore, Bangladesh

**DOI:** 10.1155/2020/2109673

**Published:** 2020-05-19

**Authors:** G. K. Acherjya, M. Ali, A. B. M. S. Alam, M. M. Rahman, S. G. M. Mowla

**Affiliations:** ^1^Jashore Medical College and Hospital, Jashore, Bangladesh; ^2^National Institute of Cancer Research and Hospital, Mohakhali, Dhaka, Bangladesh; ^3^Dhaka Medical College and Hospital, Dhaka, Bangladesh

## Abstract

**Background:**

Acute poisoning is a common scenario in the emergency department of any general hospital globally, but its pattern may vary in different parts of the world and even may be a different regional variation in the same country.

**Objective:**

Our recent study aims to assess the demographic characteristics, psychological aspect, pattern, and treatment outcome in different acute poisoning.

**Method:**

The present cross-sectional study was conducted in the medicine department of Jashore Medical College and Hospital from 1^st^ January to 30^th^ June 2018, which recruited 487 eligible cases of admitted acute poisoning patients.

**Results:**

The study reveals that the total incidence of acute poisoning in Jashore, Bangladesh, is 17.1 per 100,000 populations over a 6-month period. The mean age of our study population was 27 ± 11 (SD) years with having significant female preponderance in acute poisoning (female: 253/52% and male: 234/48%; *p* = 0.002). Female subjects were significantly younger than male (*p* <0.001). Moreover, the total suicidal intension of acute poisoning in our study was 97.3%, whereas the female subjects were more committed to suicidal attempts (*p* = 0.027). Organophosphorus compounds (OPCs) were the significant leading agents (66.1%, *p* = 0.029) of acute poisoning, and even, it had been significantly used as suicidal intention of poisoning substance (65.1%, *p* <0.001) in our observation. Muslim (97.5%, *p* = 0.005), 10–29 year age group (68.0%, *p* = 0.002), rural (99.2%), unmarried (51.3%), middle class (50.1%), students (48.9%), and secondary educational background population (76.4%) were more victimized of acute poisoning. Among different factors, familial disharmony constituted of 56.1% cases of suicidal attempt in acute poisoning. Finally, we had observed that the death incidence by acute poisoning in Jashore, Bangladesh, was 1.9 per 100,000 population over a 6-month period.

**Conclusion:**

The recent study reveals that there is high incidence of acute poisoning in Jashore, Bangladesh, with a significant amount of death toll. Organophosphorus compound is the most common agent of deliberating self-poisoning in our study due to its easy availability in our agriculture-based community.

## 1. Introduction

A poison administered by any route is capable to produce ill health, disease, or death [1]. Acute poisoning is a major public health problem worldwide with significant morbidity and mortality in all age and sex groups. This is more common in the low- and middle-income countries due to socioeconomic factors, cultural diversity, development of agricultural activities, and the promotion of agrochemicals [2]. Generally, children are more vulnerable to accidental poisoning, whereas the young adults are more committed to suicidal poisoning attempts [3]. Pesticide self-poisoning is a major global health burden which is particularly prevalent in South East Asia. There is a regional variation in the rate of suicides by pesticide self-poisoning from 0.9% in low- and middle-income countries in the European region to 48.3% in low- and middle-income countries in the Western Pacific region [4]. About 4.8 million healthy lives per annum are deceased by unintentional poisoning where pesticides play a significant role [5]. Bangladesh is one of the densely populated developing agriculture-based countries in South East Asia. There is increasing incidence of acute poisoning related to death and hospital admission in our country due to rapid development of agrochemicals and their easy availability in the community. Study has reported that pesticides are the commonest chemical agents used for acute poisoning in our country, whereas additive drugs are next to the insecticides [6]. Moreover, the commonly used substances for acute poisoning in our country are pesticides, insecticides, sedative drugs, copper sulphate, kerosene, rat killer, toilet cleaner, and nail polish. In self-poisoning, psychiatric illness plays a crucial role which always remains hidden during history documentation and management of poisoning cases. Patient's education and treatment of the underlying psychiatric illness may be a strategy which is scarcely discussed in acute poisoning where the psychiatrist may play a vital role to prevent further deliberating self-harm [5, 6]. The incidence of mortality and morbidity of acute poisoning depends on the several factors, but the early detection and prompt management of the critically ill poisoned patients are the key components. With the view of this concept, we have designed this cross-sectional study to assess the pattern, demographic characteristics, psychological aspect, and treatment outcome of different acute poisoning in the district of Jashore, Bangladesh.

## 2. Methodology

### 2.1. Study Design and Duration

The present cross-sectional study recruited all eligible 487 cases of admitted acute poisoning patients from 1^st^ January to 30^th^ June 2018.

### 2.2. Study Area

The study area is medicine department of Jashore Medical College and Hospital, Bangladesh.

### 2.3. Patient Selection Criteria

The patients with snake bite, electrocution, drowning, food poisoning, and allergic reaction due to drugs were excluded from the study. The patient's attendants who were unwilling to give informed written consent to use their data were also excluded from the study. Detailed history and relevant clinical examination data were collected from all the participated cases. The poisoning cases demonstrating on the basis of patient's statement, statement of the witness, smell of poisonous agents, and characteristics signs and symptoms of poisoning were recorded in the data sheet form.

### 2.4. Data Collection and Storage

A structured questionnaire was developed to collect the data from hospital admitted patients. The preformed data sheet included the following:

- Demographic characteristics: age, sex, religion, marital status, residence, educational status, occupational status, and monthly income in BDT
- Poison related: type of poisoning and intension of poisoning
- Data related to suicidal attempts: causes of suicidal attempts, previous history of suicidal attempts, and previous history of documented psychiatric illness
- Data related to management: treatment received prior to hospital admission and outcome of treatment after admission in the studied hospital

### 2.5. Management

After taking proper history and completion of physical examination, all the patients were treated with standard protocol. In some cases, relevant investigations such as complete blood count (CBC), random blood sugar (RBS), liver function test (LFT), renal function test (RFT), prothrombin time, international normalized ratio (INR), serum electrolytes, and chest X-ray were done to see the complications and prognosis of the patients.

### 2.6. Ethical Clearance

Institutional approval was taken from superintendent of Jashore Medical College and Hospital, Bangladesh. Personal information of patient's privacy was not disclosed to any third party. Informed written consent was taken from every case before analysis.

### 2.7. Statistical Analysis

Analysis was carried out using SPSS version 23. Categorical data were grouped as percentages and mean with standard deviation (SD) measured from continuous data. Chi-square, independent *t*-test, and one-way analysis of variance (ANOVA) used to extract the *p* value, and Games–Howell post hoc test was used to evaluate difference between different groups.

## 3. Results

This cross-sectional study was conducted with 487 eligible cases in the Medicine Department of Jashore Medical College and Hospital, Bangladesh. Mean age of study cases is 27 years with a standard deviation of 11 years. Female subjects were highly significantly younger than male subjects in this analysis (25 ± 9 vs 29 ± 13 years, *p*  < 0.001). Maximum incidence of acute poisoning was observed in people aged between 10 and 29 years, and females were significantly more than males (52.0% vs 48.0%, *p*  < 0.002), but in case of older subjects, males were more than females (Table 1).

**Figure 1:**
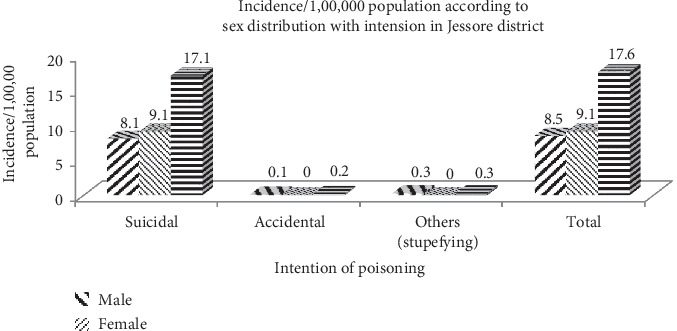
Incidence per 100,000 population according to age and sex distribution in Jashore district (Report of Department of Medicine, Jashore Medical College, Jashore) in a 6-month period. Total population of Jashore is 2764547, according to the 2011 census, Jashore district.

In this analysis, we had found that most cases (97.3%) were committed to suicidal intension of acute poisoning, whereas females were significantly (51.5%, *p*=0.027) more than males among the suicidal cases. There were no observed homicidal cases here, as no case was proved to be homicidal at the time of data collection. Total incidence of acute poisoning in Jashore is 17.1 per lakh population over a 6-month period from January 2018 to June 2018, and females were more than males in acute poisoning (Table 2 and Figure 1).

Using organophosphorus compound (OPC) was the significant leading (66.1%, *p*  =  0.029) agent found in our observation for acute poisoning and most of our cases from rural residence (99.2%). Other poisoning agents were included such as sedatives (5.3%), rat killer (7.0%), kerosene (4.1%), copper sulphate (CuSO_4_) (6.2%), Harpic/phenol (6.0%), alcohol (2.7%), travel-related poisons (1.6%), paracetamol (0.6%), and antidepressant/antipsychotic (0.4%) (Tables 3).

Regarding demographic distribution of study subjects, most of the cases were from rural area of Jashore (76.4%), and among the study subjects, most of the cases had an educational background of secondary level (92%). Acute poisoning had been observed mostly among the students (48.9%) and house wives (28.3%). Most of the cases were from Muslim society (97.5%), and we found married (48.3%) and unmarried (51.3%) subjects were equally distributed in acute poisoning. However, the middle-income groups were more victimized of acute poisoning in our recent study (Table 4).

However, a significant amount of more than two-thirds of the acute poisoning patients (73.3%, *p*=0.043) had been recovered without having any complications. Among the cases of acute poisoning, 53 patients (10.9%) died of acute poisoning and the total incidence of death in acute poisoning observed in Jashore was 1.9 per 1,00,000 population in this 6-month period from January 2018 to June 2018. Only 31 cases (6.4%) were referred to other tertiary care hospitals outside Jashore. The study had shown that 9.0% of the acute poisoning patients had been absconded (Table 5).

In suicidal intention, OPC had been used significantly high (65.1%) than other agents in acute poisoning, whereas OPC was found in accidental form only in 5 cases (1%). Other agents used for suicidal attempt were sedatives, rat killer, kerosene, CuSO_4_ (copper sulphate), Harpic/phenol, alcohol, and travel-related poisons (Table 6).

In the evaluation of cause of suicidal attempts, we had found that familial disharmony played a vital role in more than half (56.1%) of the cases. Other leading causes of suicidal attempts were failure in examination (8.2%) and affair (13.3%). Economical loss also played role in 11 cases (2.3%). In 89 cases (18.8%), participants were reluctant to give such history of their suicidal attempt. There was no previous history of documented psychiatric illness, but 0.4% (2) cases had previous history of suicidal attempt in our study (Table 7).

## 4. Discussion

Acute poisoning is one of the most common causes of emergency hospital admission, whereas the patients with minor symptoms and asymptomatic cases may not seek the health care service from the hospital and they might be missed in the statistics. In Bangladesh, all the poisoning patients are remarked as a police case during their admission in the government hospital. So, the poisoning patients from the affluent family may seek their necessary treatment from the private health care settings. Therefore, the exact incidence of acute poisoning may not be found in the meanwhile even though the total incidence of acute poisoning is 17.1 per 100,000 populations over a 6-month period from January 2018 to June 2018 in this study. The last census of Bangladesh including Jashore was conducted in 2011, so we have calculated our incidence according to the census 2011 in Bangladesh. This result could not be compared to other studies from Bangladesh due to lack of regarding data, but the incidence rate of acute poisoning is very high in Srilanka [7] and Thailand [8]. We have conducted the study among 10 and above age groups with the mean age of our participants being 27 with the standard deviation of 11 years.

In our study, most of the acute poisoning cases (331, 68%) come from the second and third decade age groups and the result is very similar to the other studies [7, 9, 10]. The female gender has been more victimized from acute poisoning than that of the male group, and the younger women are affected more in this arena. The possible reasons behind this are due to emotional liability, cultural belief, and social circumstances. Some studies have reported female preponderance of deliberated self-poisoning in Srilanka and Zimbabwe similar to our finding [7, 11]. On the contrary, the older male subjects have dominated the present study in our analysis as a result of reduced working capacity, financial instability, psychological stress, and familial negligence. Generally, many studies have shown the male domination of acute poisoning in their analysis, which is similar to this observation [9, 10, 12, 13].

Acute poisoning cases have got themselves admitted through the 24 hours emergency department of Jashore Medical College and Hospital during our time frame recruited in the present study. We have excluded snake bite from our study as it seldom occurs in the Jashore. We have seen the common forms of poisoning in this area are OPC, rat killer, phenol/Harpic/nail polish remover, CuSO_4_, sedatives, kerosene, alcohol, travel-related poisoning, ingestion of paracetamol, and antidepressant/antipsychotic prior to conduct the study. There are many studies that have reported that organophosphorus compounds occupy the leading cause of acute poisoning [4, 5, 7, 9, 14]. This is due to easy availability of OPC in our society of agriculture-based country; even WHO has reported previously that use of pesticides is the most popular way of deliberating self-poisoning worldwide, and it is estimated about one-third of the global suicides is resulting from the pesticides self-poisoning [15].

The common motive of acute poisoning in our study is due to suicidal intention which constituted 97.3%, whereas the accidental and stupefying intention fills the rest of the intention. The same result of suicidal intention in the acute poisoning has been reported by a study conducted in Kathmandu, Nepal [16]. Some other studies have also reported that suicidal intention is a higher motive of acute poisoning presentation with different frequencies in their study [9, 13], but the variation in frequency of suicidal motive is possibly due to the sampling method of the their study. In our study, suicidal intention is more among the females than that of the males. This result is supported by the other analysis conducted in India and Ilam Province of West Iran [17, 18].

There are various reasons related to deliberate self-harm acute poisoning including familial disharmony, failure in affair, examination and success in career, sexual abuse, and chronic persistent illness. Out of these, familial disharmony has constituted 56.1% cause of suicidal attempts in acute poisoning cases. Many other studies have reported that familial conflict is the main cause of acute poisoning in their analysis like our study [19, 20]. In our study, 18.8% patients have not shown any interest to admit their reason of deliberating self-harm possibly due to the cultural belief and social stigmata. There is no previous history of documented psychiatric illness in our patients, but 0.4% patients have previous suicidal attempt prior to committed acute poisoning in the present study.

Although it is not statically significant in our study, we have found that deliberating self-poisoning is more common among the rural population. It has also been also shown by the another study conducted in Sylhet, Bangladesh [12].

Our study reveals that 73.7% patients have recovered from acute poisoning, whereas only 6.4% patients have been referred for better management as some of them have required intensive care unit (ICU) management which is not available in Jashore. On the contrary, the death rate in our study has 10.9% and the death incidence by acute poisoning reveals only 1.9 per 100,000 populations over a 6-month period in Jashore. One study has reported that 4% patients have died of acute poisoning even with the feasibility of ICU support in tertiary teaching hospital in Scotland [21].

## 5. Limitation

It is a 6-month study, and the incidence was carried out from 6 months' data. So, yearly incidental reflection was not carried out. The incidence of acute poisoning and death rate has calculated according to our last census 2011, Bangladesh. Moreover, the strength of this study includes demographic characteristics, pattern of acute poisoning, and treatment outcome of acute poisoning.

## 6. Conclusion

Acute poisoning still remains the major public health problem in the hospital emergency departments in many countries like Bangladesh. The recent study reveals that there is high incidence of acute poisoning in Jashore, Bangladesh, with significant amount of death toll. The younger aged populations are more vulnerable, so the awareness programs should be carried out about the toxicity, fatality, and mortality of acute poisoning. As OPC is the most common form of deliberating self-harm poisoning for suicidal motive, we recommend limiting its use with caution. Familial disharmony is the main cause of suicidal attempt in our study; we suggest improving the familial bondage to check the critical emotion.

## Figures and Tables

**Table 1:** Age and sex distribution of poisoning (*n* = 487).

Age	Sex of study subjects		Total,	*p* value
	Male	Female		
Age group	*n* (%)	*n* (%)	*n* (%)	
10–19	56 (11.5)	86 (17.7)	142 (29.2)	**0.002** ^**s**^ ** (chi-square)**
20–29	94 (19.3)	95 (19.5)	189 (38.8)	
30–39	34 (7.0)	48 (9.9)	82 (16.8)	
40–49	26 (5.3)	17 (3.5)	43 (8.8)	
50–59	12 (2.5)	5 (1.0)	17 (3.5)	
60–69	11 (2.3)	1 (0.2)	12 (2.5)	
≥70	1 (0.2)	1 (0.2)	2 (0.4)	

Total	**234 (48.0)**	**253 (52.0)**	**487 (100.0)**	

Mean ± SD	**29** **±** **13**	**25** **±** **9**	**27** **±** **11**

Min-max	**10–70**	**12–72**	**10–72**	**<0.001** ^**s**^ ** (*t*- test)**

s: statistically significant (*p* value: ≤ 0.05)

**Table 2:** Intension of poisoning according to sex (*n* = 487).

Intension	Male, *n* (%)	Female, *n* (%)	Total, *n* (%)	*p* value
Suicidal	223 (45.8)	251 (51.5)	474 (97.3)	**0.027** ^**s**^ ** (chi-square)**
Accidental	4 (0.8)	1 (0.2)	5 (1.0)	
Others (stupefying)	7 (1.4)	1 (0.2)	8 (1.6)	

Total	**234 (48.0)**	**253 (52.0)**	**487 (100.0)**	

s: statistically significant (*p* value: ≤ 0.05)

**Table 3:** Types of poisoning according to sex (*n* = 487).

Types of poisoning agent	Male, *n* (%)	Female, *n* (%)	Total, *n* (%)	*p* value
Organophosphorus compound (OPC)	163 (33.5)	159 (32.6)	322 (66.1)	**0.029** ^**s**^ ** (chi-square)**
Sedatives	9 (1.8)	17 (3.5)	26 (5.3)	
Rat killer	18 (3.7)	16 (3.3)	34 (7.0)	
Kerosene	8 (1.6)	12 (2.5)	20 (4.1)	
CuSO_4_	12 (2.5)	18 (3.7)	30 (6.2)	
Harpic/phenol	7 (1.4)	22 (4.5)	29 (6.0)	
Alcohol	7 (1.4)	6 (1.2)	13 (2.7)	
Travel-related poisoning	7 (1.4)	1 (0.2)	8 (1.6)	
Paracetamol	1 (0.2)	2 (0.4)	3 (0.6)	
Antidepressant/antipsychotic	2 (0.4)	0 (0.0)	2 (0.4)	

Total	**234** (48.0)	**253** (52.0)	**487** (100.0)	

s: statistically significant (*p* value: ≤ 0.05)

**Table 4:** Demographic distribution of study subjects attending Jashore Medical College Hospital according to intention of poisoning (*n *= 487).

Demographic characteristics		Intention of poisoning			Total, *n* (%)	*p* value
		Suicidal, *n* (%)	Accidental, *n* (%)	Others (stupefying), *n* (%)		
Residence	Rural	470 (96.5)	5 (1.0)	8 (1.6)	483 (99.2)	0.946^ns^
	Urban	4 (0.8)	0 (0.0)	0 (0.0)	4 (0.8)	

Education	Illiterate	37 (7.6)	0 (0.0)	0 (0.0)	37 (7.6)	0.952^ns^
	Primary	73 (15.0)	1 (0.2)	2 (0.4)	76 (15.6)	
	Secondary	362 (74.3)	4 (0.8)	6 (1.2)	372 (76.4)	
	Graduate/above	2 (0.4)	0 (0.0)	0 (0.0)	2 (0.4)	

Occupation	Student	231 (47.5)	4 (0.8)	3 (0.6)	238 (48.9)	0.320^ns^
	Housewife	137 (28.1)	0 (0.0)	1 (0.2)	138 (28.3)	
	Farmer	69 (14.2)	1 (0.2)	2 (0.4)	72 (14.8)	
	Service worker	9 (1.8)	0 (0.0)	0 (0.0)	9 (1.8)	
	Business	28 (5.7)	0 (0.0)	2 (0.4)	30 (6.2)	

Religion	Muslim	463 (95.1)	4 (0.8)	8 (1.6)	475 (97.5)	0.005^ns^
	Hindu	6 (1.2)	0 (0.0)	0 (0.0)	6 (1.2)	
	Christian	5 (1.0)	1 (0.2)	0 (0.0)	6 (1.2)	

Marital status	Married	226 (46.4)	3 (0.6)	6 (1.2)	235 (48.3)	0.618^ns^
	Unmarried	246 (50.5)	2 (0.4)	2 (0.4)	250 (51.3)	
	Separated/widow	2 (0.4)	0 (0.0)	0 (0.0)	2 (0.4)	

Monthly income in BDT	<5000	99 (20.3)	2 (0.4)	1 (0.2)	102 (20.9)	0.696^ns^
	5000–10000	91 (18.7)	1 (0.2)	3 (0.6)	95 (19.5)	
	10000–15000	238 (48.9)	2 (0.4)	4 (0.8)	244 (50.1)	
	>15000	46 (9.4)	0 (0.0)	0 (0.0)	46 (9.4)	

Total		**474 (97.3)**	**5 (1.0)**	**8 (1.6)**	**487 (100.0)**	

ns: statistically not significant, (*p* value significant : ≤ 0.05)

**Table 5:** Treatment outcome in relation to intention of poisoning of the study patients (*n* = 487).

Intention	Outcome of treatment					Total	*p* value
	Recovered	Absconded	Referred^*∗*^	Death			
	*n* (%)	*n* (%)	*n* (%)	*n* (%)	Per 100,000		
Suicidal	353 (72.5)	42 (8.6)	29 (6.0)	50 (10.3)	1.8	474 (97.4)	0.043^s^
Accidental	3 (0.6)	0 (0.0)	0 (0.0)	2 (0.4)	0.1	5 (1.0)	
Others (stupefying)	3 (0.6)	2 (0.4)	2 (0.4)	1 (0.2)	0.0	8 (1.6)	

Total	**359 (73.7)**	**44 (9.0)**	**31 (6.4)**	**53 (10.9)**	**1.9**	**487 (100.0)**	

s: statistically significant (*p* value: ≤ 0.05)

**Table 6:** Types of poisoning in relation to intention of poisoning of the study patients (*n* = 487).

Types of poisoning	Intention of poisoning of the study patients				*p* value
	Suicidal, *n* (%)	Accidental, *n* (%)	Others (stupefying), *n* (%)	Total	
Organophosphorus compound (OPC)	317 (65.1)	5 (1.0)	0 (0.0)	322 (66.1)	<0.001^s^
Sedatives	26 (5.3)	0 (0.0)	0 (0.0)	26 (5.3)	
Rat killer	34 (7.0)	0 (0.0)	0 (0.0)	34 (7.0)	
Kerosene	20 (4.1)	0 (0.0)	0 (0.0)	20 (4.1)	
CuSO_4_	30 (6.2)	0 (0.0)	0 (0.0)	30 (6.2)	
Harpic/phenol	29 (6.0)	0 (0.0)	0 (0.0)	29 (6.0)	
Alcohol	13 (2.7)	0 (0.0)	0 (0.0)	13 (2.7)	
Travel-related poisoning	0 (0.0)	0 (0.0)	8 (1.6)	8 (1.6)	
Paracetamol	3 (0.6)	0 (0.0)	0 (0.0)	3 (0.6)	
Antidepressant/antipsychotic	2 (0.4)	0 (0.0)	0 (0.0)	2 (0.4)	

Total	**474 (97.3)**	**5 (1.0)**	**8 (1.6)**	**487 (100.0)**	

s: statistically significant (*p* value: ≤ 0.05)

**Table 7:** Causes of suicidal attempts (*n* = 474).

Causes of suicidal attempts	*n* (%)
Familial disharmony	266 (56.1)
Failure in examination	39 (8.2)
Failure in affair	63 (13.3)
Economical loss	11 (2.3)
Chronic illness	2 (0.4)
Adultery/sexual abuse	2 (0.4)
Reluctant to give such history	89 (18.8)
Previous history of suicidal attempt	2 (0.4)
Previous history of documented psychiatric illness	0 (0.0)

## Data Availability

The data used to support the findings of this study are available from the corresponding author upon request.
